# Supplementary material for: ‘Just Play’ (JP) - creative arts therapies-based dyadic intervention for children with intellectual disability and their mothers: Study protocol for a mixed-methods randomized controlled trial
Source: PLoS One. 2026 May 21;21(5):e0349576. doi: 10.1371/journal.pone.0349576 (PMC13193551; doi:10.1371/journal.pone.0349576)
Supplement: S2 File — (DOCX) [file pone.0349576.s002.docx]

**To: Faculty Research Ethics Committee**

**Application for Approval of Study**

**Title of research proposal: Dyadic Creative Arts Therapy Intervention for Parents and Children with Intellectual and Developmental Disabilities**

______________________________________________________________________________

**Date:** 07/12/2022 ____________________________________________________________________________

**By:** Dr. Rinat Feniger-Schaal

**Mobile phone number:** 052-606-0228

**Email address:** [rinat@feniger.com](mailto:rinat@feniger.com)

______________________________________________________________________________

**Department / school (of Investigator A):** The School of Creative Arts Therapies ______________________________________________________________________________

**Name of additional investigator:** Prof. Cochavit Elefant

**Mobile phone number:** 054-594-4158

**Email address:** [celefant@univ.haifa.ac.il](mailto:celefant@univ.haifa.ac.il)

______________________________________________________________________________

**Department / school (of Investigator B):** The School of Creative Arts Therapies

______________________________________________________________________________

**Name of additional investigators and students:**  Prof. Limor Goldner, Dr. Tal-Chen Rabinowitz, Rita Abramov and Amitai Stern

**Mobile phone number:** Press or type here to enter text

**Email address:** [limor.goldner@gmail.com](mailto:limor.goldner@gmail.com), [talchenr@gmail.com](mailto:talchenr@gmail.com) , rita.abramov@gmail.com, amitaistern1@gmail.com

______________________________________________________________________________

**Department / school** The School of Creative Arts Therapies

______________________________________________________________________________

1. **General**

**If applicable - Please check:**

**Request for exemption from Ethics Committee proceeding**

**Reason for request:** Press or type here to enter text

**X Request for Expedited review proceeding**

**Reason for request:** The investigators wish to perform a pilot study in order to better understand the needs of the given population and to adapt the planned intervention before proceeding to the following stages of the research.

**Request for Exemption from participants' written consent requirements**

**Reason for request:** Press or type here to enter text

- 1. **If the research is carried out as part of studies for a master's / doctoral degree –**

**has the approval of the departmental / school committee for the master's / doctoral program been obtained?**  Yes **X** No

**2. Concise description of the study (up to 200 words)**

The proposed research aims to design and evaluate the contribution of a dyadic Creative Arts Therapy (CAT) intervention for children with Intellectual and Developmental Disabilities (IDD) and their parents. By engaging playfully in a spontaneous play in a non-judgmental and pleasurable space through the proposed intervention, we believe that children with IDD and their parents will increase the quality of their play and mutual interactions, consequently strengthening the parent-child relationship. The proposed study will apply a randomized controlled trial (RCT) mixed-methods, longitudinal study design that will include sixty parent-child dyads of one parent and a child with IDD. 30 dyads will be randomly assigned to the intervention condition and 30 dyads will be assigned to the control condition. The intervention group (n=30) will receive an 8-week dyadic play intervention and the control group (n=30) will receive an 8-week psycho-educational counseling. The mixed-methods will combine quantitative assessments (before and after the interventions) of the Emotional Availability (EA) scales, parent’s playfulness (Parent Playfulness Scale), child playfulness (ToP) and parent-child Interpersonal Synchrony (IPS), together with a qualitative assessment that will interview a subsample of ten parents during the final assessment meeting and two months after the intervention ends. The full protocol of the dyadic CAT intervention will be developed as part of the study. The first stage of the study will include a pilot, in which two dyads of a parent and a child (n=2) with IDD will receive a shortened version of the planned dyadic CAT intervention) in order to better understand the needs of the given population and to adapt the planned intervention. An expedited ethical review will be appreciated in order to begin the pilot study promptly.

**3. General evaluation of risk in the study (please check only one):**

To the best of my knowledge, the proposed study poses no risk of harm to participant/s or their surroundings.

**X** It is my opinion that the extent of risk to participants in the proposed study is less than minimal risk and the requisite measures to mitigate said risk have been taken.

- “Minimal risk:” The severity and/or probability of risk of harm or discomfort expected in the study do not exceed those to which a reasonable person is exposed in his/her daily conduct or in the course of taking routine psychological or physical exams or checkups.

It is my opinion that the level of risk to participants in the proposed study exceeds the minimal risk, and the requisite measures have been taken to protect the participant/s to the greatest extent possible.

**4. Research Participants**

**4.1 Number of participants:** 60 parent-child dyads resulting in a minimum of 120 participants

|  | |  |  | **Yes** | **No** | **Comments / details** | |  |
| --- | --- | --- | --- | --- | --- | --- | --- | --- |
| **4.2** | | **Age range**: | | Minors (under age 18)—a parental consent form and, if the minor is an adolescent, his/her assent is needed | **X** |  | The parents will consent to their own participation in the study, as well as their children’s participation. The children will be pre-adolescent so their consent will not be necessary. The children will participate only in the two assessment meetings, as well as in the dyadic play section of the CAT intervention. The other elements of the research method involve only parents and their giving consent to participating. | |
| **4.3** | | **Type of population** | | Pupils / their parents, recruited via the educational system | **X** |  | Participants for the pilot study will be recruited through posted flyers on social media pages, websites of organizations and associations dedicated to people with IDD and parents’ support groups. Participants for the full study will be recruited through the educational frameworks, upon approval of the Ministry of Education ethics committee. | |
|  | |  | Students at the University of Haifa and/or members of their families |  | **X** |  |  |  |
|  | |  | Adult population without weaknesses |  | **X** |  |  |  |
|  | |  | Sensitive population groups (e.g., wards, prisoners, individuals with cognitive disorders; mental illness, etc.) | **X** |  | The participants will be children with IDD and their parents. |  |  |

**4.4 Participants' recruitment Process (explain in detail, including how they are located, screened, by whom contacted, in what manner, etc.)**

Participants for the pilot study will be recruited through posted flyers on social media pages, websites of organizations and associations dedicated to people with IDD and parents’ support groups. Participants for the full study will be recruited through the educational frameworks, dependent upon approval of the Ministry of Education ethics committee.

**5. Please indicate whether the study includes one or more of the following research methods. If answering in the affirmative, please provide a detailed explanation in the study abstract.**

|  |  | **Yes** | **No** |
| --- | --- | --- | --- |
| **1.** | **Questionnaire (participants identified to investigator)** | **X** |  |
| **2.** | **Anonymous survey (participants not identified to investigator)** |  | **X** |
| **3.** | **Interview** | **X** |  |
| **4.** | **Observation** | **X** |  |
| **5.** | **Video or camera recording, etc. (if “yes” please specify below how it will be saved)** | **X** |  |
| **6.** | **Audio recording (if “yes,” please specify below how it will be saved)** | **X** |  |
| **7.** | **Use of existing documents or data (including information from medical records, databases, etc.)** |  | **X** |
| **8.** | **Structured tests (e.g., behavioral test or task)** | **X** |  |
| **9.** | **Experimental set-up (experimental manipulation)** | **X** |  |
| **10.** | **Collection of biological samples** |  | **X** |

**5.1 If the study includes the use of biological samples, audio and/or video and/or transcribed interviews, explain in detail whether and how the data will be stored, when will it be deleted, whether it will be returned to the participants or shared with them, and how anonymity/confidentiality will be preserved in this regard:**

The study will involve video and audio recordings, questionaries and transcribed interviews. The data will be stored securely on an organizational Google drive account. The privacy and anonymity of the participants will be protected, and they will be identified in the video, audio and interview data only by code numbers. The participants’ identities and personal data will be stored in a separate file on the primary researchers’ computers secured with a password in order to prevent the video recordings from being identified. The materials will be kept for 7 years for the research purposes and will subsequently be destroyed.

**6. Please indicate whether the study includes one or more of the following elements (please provide details in the right-hand column):**

|  |  | **Yes** | **No** | **Comments** |
| --- | --- | --- | --- | --- |
| **1.** | **Misleading or inadequate explanation** |  | **X** |  |
| **2.** | **Collection of sensitive information** |  | **X** |  |
| **3.** | **Exposure to stimuli that may be experienced as threatening, insulting, triggering anxiety, triggering traumatic memories, etc.** |  | **X** |  |
| **4.** | **Exposure to physical stimuli (e.g., high levels of noise, pain or visual stimuli that exceed routine daily levels of irritation)** |  | **X** |  |
| **5.** | **Collection of biological and/or physiological indicators (e.g., blood, saliva, pulse, blood pressure, other physiological indicators)** |  | **X** |  |
| **6.** | **Use of pharmaceuticals (describe the pharmaceuticals and the measures taken to maintain participants’ safety)** |  | **X** |  |
| **7.** | **Physical effort exceeding accepted daily levels (describe the task and the measures taken to protect participants)** |  | **X** |  |
| **8.** | **Social, legal, or economic risk to participants (e.g., creation of stigma, risk to status, risk to employment, or criminalization of participants)** |  | **X** |  |
| **9.** | **Recruitment of participants via persons of authority (teacher, caregiver, employer)** |  | **X** |  |
| **10.** | **Monetary recompense, academic grades, or other means of encouraging participants (describe in the Comments)** |  | **X** |  |

**6.1 If you answered “yes” to any of the above, please detail here in what way/s the study will deal with the ethical complexity or the potential damage of the cited element:**

Participating in the dyadic intervention and the control group may evoke feelings, thoughts and memories among the parents participating in regard to their parenthood, especially those related to their parenthood to a child with disabilities. The research team is composed of qualified therapists with experience in work with children with disabilities and they will provide support and assistance to the participants as an integral part of the interventions proposed. In addition, the research team will provide parents with their contact information in case a need for an additional support arises, as well as actively inquire at given points during the intervention process whether the parents feel in need of any further support.

**6.2 If you answered “yes” to any of the above, please elaborate on the relevant investigators’ training and background for dealing with the ethical complexity of the cited element.**

The researchers are qualified, experienced therapists with previous experience working with children with IDD and with parents.

**7. If an exemption from signing consent forms is requested, how shell participants' consent be obtained / documented?**

Press or type here to enter text

**8. Please indicate whether the following are included in the consent form and/or explanatory letter to potential participants (if “no”—explain why not in the Comments section)**

|  |  | **Yes** | **No** | **Comments** |
| --- | --- | --- | --- | --- |
| **1.** | **Description, title, and purpose of the study. (Please note if the study is part of a seminar, a thesis, or a dissertation.)** | **X** |  |  |
| **2.** | **Benefit/s of the study** | **X** |  |  |
| **3.** | **Side effects or risks to the participant** | **X** |  |  |
| **4.** | **Tasks assigned to the participant/s** | **X** |  |  |
| **5.** | **Affirmation of voluntary participation and participants’ right to withdraw from the study at any time without personal consequences** | **X** |  |  |
| **6.** | **Assurance of confidentiality, anonymity, and privacy (including how the data is retained and destroyed in cases of research on identified persons)** | **X** |  |  |
| **7.** | **Expected duration of participation in the study** | **X** |  |  |
| **8.** | **Source of study funding (if external to the university)** | **X** |  |  |
| **9.** | **Name of investigator and telephone number or other contact information. (If the study is part of a thesis or dissertation, advisors’ names should be noted as well.)** | **X** |  |  |
| **10.** | **Voluntary participation consent form** | **X** |  |  |
| **11.** | **Verification of participant's signature in presence of investigator** | **X** |  |  |

**9. Safeguarding of confidentiality of collected data**

|  |  | **Yes** | **No** | **Comments** |
| --- | --- | --- | --- | --- |
| **1.** | **Will consent forms be kept separate from participants’ data?** | **X** |  |  |
| **2.** | **Will participants’ identities be disclosed in publications?** | **X** |  |  |
| **3.** | **Will Identified/identifiable details be available to someone other than the members of research team?** | **X** |  |  |

**9.1 Please describe the measures that will be taken to protect participants’ identities and secure the data obtained / collected:**

The data will be stored securely on an organizational Google drive account. The privacy and anonymity of the participants will be protected, and they will be identified in the video, audio and interview data only by code numbers. The participants’ identities and personal data will be stored in a separate file on the primary researchers’ computers secured with a password in order to prevent the video recordings from being identified. The materials will be kept for 7 years for the research purposes and will subsequently be destroyed.

**I hereby affirm that the foregoing information is correct and accurate, that the research proposal complies with international and the university’s standards for ethical research conduct, and that the study will be carried out in accordance with said standards.**

**I am aware that the responsibilities of the chief investigator include reviewing the ethical guidelines and conduct of the different entities involved in the study's execution (e.g., students engaged in the study, research assistants, information-gathering companies, various professional entities, etc.). The ethical guidelines and conduct of these bodies are not explicitly reviewed by the Ethics Committee.**

**Dyadic Creative Arts Therapy Intervention for Parents and Children with Intellectual and Developmental Disabilities**

1. **Background**

Play is considered one of the most crucial components that contribute to children’s healthy development and through which their socioemotional abilities evolve (Frost, 2010). Children with intellectual and developmental disabilities (IDD) experience cognitive difficulties that impair their ability to play, explore and communicate with the environment (American Psychiatric Association [APA], 2013), thus inhibiting the development of their playing skills. The research on the play of children with IDD is limited, with existing works showing that the play patterns of children with IDD tend to be more repetitive, lacking in creativity and involvement with peers (Astramovich et al. 2015). These deficiencies profoundly influence the social development of children with IDD and may lead to long-lasting emotional and behavioral problems. Thus, early interventions are often proposed to promote playfulness among children with IDD and ensure improved developmental outcomes (Mahony et al. 1998).

In the last few decades, the early intervention practice has moved in the direction of family-centered models, focusing on assisting parents to develop skills that promote better interpersonal engagement between them and their children (Gilboa & Roginsky, 2010). This shift is based on ample research showing that children’s cognitive social-communicative development is directly related to the nature of parent-child interactions (Connell & Prinz, 2002; Dodici et al. 2003). Positive interactional experiences with parents in early childhood that include parental responsiveness, sensitivity and warmth were found among the most critical indicators of children’s development (Baker et al. 2005; Feniger Schaal et al. 2012). Furthermore, recent pioneering studies that have examined the effect of parental playfulness on parents’ interaction with their children and the children’s adjustment skills, found that parental playfulness, when joyfulness, fun and creativity are present in the parent-child interactions, can lend parents a unique opportunity to establish a positive communication approach with their children (Menashe‐Grinberg & Atzaba‐Poria, 2017). As of today, not much research has been done to investigate parental playfulness among parents of children with typical development, and even less among parents of children with IDD (Levavi et al., 2020). However, parental playfulness seems to be an essential component in the interactions between parents and children with IDD, because they tend to experience difficulties in interpersonal interaction and in verbal communication (Martin et al., 2010). Furthermore, parents of children with IDD often encounter challenges in their relationships with their children, exhibiting high stress levels, difficulties in reading their child’s signals and a general lack of joy in the parent-child interaction (Gerstein & Crnic, 2016; Norona & Baker, 2017). Thus, early dyadic interventions focusing on promoting playful interactions may contribute to the relationships of parents and children with IDD, hence improving their future developmental outcomes (Guralnick, 2017).

The proposed study suggests to create and implement an innovative dyadic intervention tailored specifically for the understudied population of children with IDD and test its effects on the quality of the parent-child relationship among children with IDD. The intervention will be based on the Creative Arts Therapies (CAT) and their unique attributes such as playfulness, multi-sensorial experiences, creativity and emotional self-expression. The dyadic CAT intervention will promote these qualities through non-verbal communication and by forming engaging and joyful moments for children with IDD and their parents, thereby enhancing the quality of their interactions and relationships.

1. **Objectives**

The objectives of this study are as follows:

- To examine whether the dyadic CAT intervention will improve the parent-child relationship among children with IDD compared to psycho-educational parent counseling, as reflected through Emotional Availability (EA) scales and parent-child Interpersonal Synchrony (IPS).
- To examine the effect of the dyadic CAT intervention on children’s and parent’s playfulness as measured by the child Test of Playfulness (ToP) and the Parental Playfulness Scale.
- To explore the parental experience within the dyadic CAT intervention and examine what intervention features the parents found most essential to stimulating playfulness and mutual engagement in interactions with their children.
- To investigate how the dyadic CAT intervention affects the daily routines of families with children with IDD after its termination (2 months later).

The study’s hypothesis is that the dyadic CAT intervention will lead to improved parental EA, parent-child IPS, and both parent’s and child’s playfulness levels, and will have a stronger positive effect than the psycho-educational parent counseling. It is predicted that the dyadic CAT intervention will enable parents to amplify joyful and engaging interactions in their daily routines with their children with IDD, and thus will positively affect their relationship quality in a way that will last after the intervention ends.

1. **Method**

**Participants:** The research will include sixty voluntary parent-child dyads of one parent and a child with IDD. The participants will be invited to take part in the study through social media posts on Facebook, websites of organizations and associations dedicated to people with IDD and parents’ support groups. Participants for the full study will be recruited through preschool educational frameworks for children with IDD, depending on the approval of the Ministry of Education ethics committee. The inclusion criteria for participation in the research will be dyads with a 3-7 years old child diagnosed with IDD. Parents will be asked to provide signed consent for their children to participate in the study.

*Age Criteria:* At their respective time of randomization, the children’s age within each dyad will range between 3 to 7 years. As the proposed study aims to target dyadic experiences in early childhood and examine their effect on the quality of the parent-child relationship, inclusion of young children in the study is necessary. The lower age boundary was chosen based due to the fact that the diagnosis of IDD is usually assessed at the age of around 3 years old. The upper age boundary was chosen in order to limit the sample to a group that shares similar everyday life conditions in preschool settings.

*Diagnosis Criteria:* Children within the participating dyads must have a prior diagnosis of a mild to moderate intellectual and developmental disability by a pediatrician, neurologist or developmental psychologist. Mild IDD is a specific intellectual disability that refers to children characterized by an intellectual and adaptive functioning 1.5-2 standard deviations below the mean and moderate IDD is 3-4 standard deviations below the mean (Patel et al., 2020).

*Exclusion criteria:* Children participating in the research must not have another primary disorder diagnosis, such as autism spectrum disorder, epilepsy, cerebral palsy or sensory disorder- such as blindness or deafness, as this might affect and alter the course, implementation and the results of the proposed study.

**Study Design:** The proposed study will apply a randomized controlled trial (RCT) mixed-methods, longitudinal study design that will include sixty parent-child dyads of one parent and a child with IDD that will participate in a baseline assessment meeting, 8 weekly sessions of either intervention or control, and a final assessment meeting. Each of these ten meetings will last approximately 60 minutes. A subsample of ten parents will be interviewed during the final assessment meeting and two months after the intervention has ended, each interview lasting approximately one hour. The first stage of the study will include a pilot, in which two dyads of a parent and a child with IDD will receive a shortened version of the planned dyadic CAT intervention that will include a baseline assessment meeting, four weekly creative arts therapies-based sessions in an individual setting and a final assessment meeting. The pilot stage of the study will enable the researchers to assess the dyadic CAT intervention protocol and make necessary adaptations for the following stages of the study.

**Procedure:** After initial contact and a comprehensive explanation concerning the study, the parents, whose children meet the eligibility criteria, will be asked to sign informed consents to participate in the study and then the baseline assessment meeting will be scheduled. The assessment meeting will be conducted by the research team at the participants’ home and will include a few parent-child playing episodes, such as a free play episode, symbolic play episode, social play episode and a mutual movement episode to a familiar children song, as well as completing a demographic questionnaire. After the assessment meeting, all parent-child dyads will be randomly assigned to the experiment group (dyadic CAT intervention) or the control group (psycho-educational parent counseling) according to a computer-generated randomization list. The allocation ratio of intended numbers of participants in the comparison groups will be 1:1 so that the number of dyads receiving each intervention will be similar. The final assessment meeting will take place one week following the last intervention, during which the research team will re-evaluate the parent-child playing interaction as in the baseline assessment meeting. The meetings will be recorded using two video cameras in order to enable capturing both the parent and the child during their interaction. In addition, parents will be asked to complete again a self-report questionnaire regarding their relationship with their children and their play perception, as well as a session evaluation questionnaire. A semi-structured in-depth interview will be conducted to a subsample of ten parents concerning their experiences during the intervention sessions at the final assessment meeting and two months after the intervention ends. The interviews will be audio recorded and then transcribed.

**Interventions:** The participating dyads will be randomly assigned to one of the following two conditions:

*(1) Dyadic CAT intervention:* creative arts therapies-based sessions in an individual setting once a week for 8 weeks. The CAT intervention sessions will include a part of a dyadic play that will engage both the parent and the child and will last 45 minutes, and a following part of discussion between the therapist and parent, lasting for another 15 minutes. All sessions will be conducted by trained creative art therapists with clinical experience with children with IDD. The dyadic play part will be video recorded in order to be used for discussion during the session analysis by therapist and parent.

*(2)* *Psycho-educational parent counseling:* counseling sessions in an individual setting once a week for 8 weeks for parents only. Each session will last approximately 60 minutes and will be conducted by trained parent counselors experienced in the field of IDD.

It should be noted that children will participate only in the two assessment meetings, as well as in the dyadic play section of the CAT intervention. The other elements of the experimental and control intervention will involve only the parents.

**Treatment guide:** The dyadic CAT intervention and the psycho-educational parent counseling will both be provided in accordance with a treatment guide developed for this study in order to specify the treatment procedures and to allow for staff training and replication of treatment. Within this guide, the specific goals, principles and exemplifications of the intervention sessions will be outlined. While the treatment guide will help to ensure the study’s validity and replicability, some flexibility and openness to emerging situations within meetings will be allowed in order to ensure that the intervention is tailored to the individual strengths and needs of each child, thus addressing the variability of developmental profiles present in children with IDD.

*Dyadic CAT intervention:* The main goal of the intervention is to provide participants with joyful and engaging dyadic interactions and promote a mutual imaginative world via multi-sensorial exploratory experiences, playfulness, creativity, imagery, and emotional self-expression through non-verbal communication, following the child's lead. As the interventions do not bear any pedagogical agenda, the focus of the sessions is the interaction and the encounter itself. Each session will contain the following elements to stimulate positive dyadic interactions: greeting and farewell songs to indicate the time limits of the session and promote repetition and familiarity; action and movement games to encourage mutual engagement, fun, creativity, turn-taking and sharing; familiar children songs and nursery rhymes that invite a rhythmic body movement (clapping, body percussion, etc.) to develop joint attention and interpersonal synchrony within the dyad; movement vocalizing to animate each other; quiet singing to encourage physical touch, intimacy and bonding between parent and child. The action and movement games will be based on basic play principles of affect exaggeration, humor, surprise and sensorimotor experiences and will include games such as peekaboo and hide-and-seek games, sensory play with a piece of cloth and imaginative games with simple toys available in the natural surroundings of the child. The dyadic play stage will be followed by discussion by the parent and the therapist, in which the session content will be analyzed and ideas for its implementation in the daily routines will be offered.

*Psycho-educational parent counseling:* Counseling sessions will comprise supporting conversations with parents, focusing on improving the parent-child relationship and dealing with concerns and difficulties arising from the child’s diagnosis, development and behavior, while also providing them with information about IDD, child development, and social communication relevant to their everyday life situations.

**Assessment of treatment fidelity:** In order to ensure that the treatment is conducted as intended, the following fidelity check measures will be applied: after each session, the therapists/counsellors will note whether they performed the meeting according to the protocol and will document significant events, notable child/parent behaviors and interventions applied during the meeting. In addition, all sessions both in the experiment and the control group will be videotaped to allow for assessment by independent raters. In addition, therapists’ and counsellors’ adherence to the method and their competence in its application will be monitored and sustained through supervision by the same supervisor, utilizing therapists’ and counsellors’ notes and video-recordings of sessions when necessary.

**Outcomes measures:** The quantitative assessments will include the following variables that will be measured before and after the interventions in both the experiment and control groups. *Emotional Availability Scale* (EAS; 4th Edition, Biringen et al., 2014) is a standardized observational video analysis tool that will assess parents’ sensitivity, structuring, non-intrusiveness, non-hostility and child’s responsiveness and involvement based on episodes of free and semi-structured play of parents with their children. *Parent-Child* *Interpersonal* *Synchrony* (IPS) will be evaluated by micro-analyzing the movement coordination between a parent and a child when moving together to a musical piece composed for the project. IPS will be analyzed with the tracking software (OpenPose) similarly to Himberg & Thompson, 2011. *Test of Playfulness* (ToP; version 4.2, Bundy, 2010) is a standardized observational video analysis tool developed to evaluate and score children’s play. Studies have established the reliability and validity of the ToP for use with children with developmental disabilities (Hamm, 2006; Okimoto et al., 2000). *Parental Playfulness Scale* (Atzaba-Poria et al., 2014) is an observational video analysis tool that assesses parental creativity, imagination, humor, pretend play and curiosity while playing with their children and has been used with children with IDD (Levavi et al., 2020). The qualitative assessment will include in depth-semi structured interviews that will assist to learn about participants’ experience throughout the intervention and examine its sustainability.

**Data management and storage:** The study will involve video, questionaries and transcribed interviews. The data will be stored securely on an organizational Google drive account. The privacy and anonymity of the participants will be protected, and they will be identified in the video and interview data only by code numbers. The participants’ identities and personal data will be stored in a separate file on the primary researchers’ computers secured with a password in order to prevent the video recordings from being identified. The materials will be kept for 4 years for the research purposes and will subsequently be destroyed.

**References**

American Psychiatric Association. (2013). *Diagnostic and statistical manual of mental disorders* (5th

ed.). Washington, DC: Author.

Astramovich, R. L., Lyons, C., & Hamilton, N. J. (2015). Play therapy for children with intellectual

disabilities. *Journal of Child and Adolescent Counseling*, *1*(1), 27-36.‏

Atzaba-Poria, N., Carbrera, N. J., Menashe, A., & Karberg, E. (2014). The parental playfulness scale. Unpublished Manuscript. Beersheba, Israel: Ben-Gurion University of the Negev.

Baker, B. L., Blacher, J., & Olsson, M. B. (2005). Preschool children with and without developmental

delay: Behaviour problems, parents’ optimism and well-being. *Journal of Intellectual Disability Research, 49*(8), 575–590.

Biringen, Z., Derscheid, D., Vliegen, N., Closson, L., & Easterbrooks, M. A. (2014). Emotional availability (EA): Theoretical background, empirical research using the EA Scales, and clinical applications. *Developmental Review, 34*(2), 114-167.‏

Bundy, A. (2010). *Test of Playfulness.* Lidcombe, NSW: University of Sydney.

Connell, C. M., & Prinz, R. J. (2002). The impact of childcare and parent-child interactions on school

readiness and social skills development for low-income African American children. *Journal of*

*School Psychology, 40*(2), 177–193.

Dodici, B. J., Draper, D. C., & Peterson, C. A. (2003). Early parent-child interactions and early literacy

development. *Topics in Early Childhood Special Education, 23*(3), 124–136.

Feniger-Schaal, R., & Oppenheim, D. (2013). Resolution of the diagnosis and maternal sensitivity

among mothers of children with intellectual disability. *Research in developmental disabilities*, *34*(1), 306-313.

Frost, J. L. (2010). *A history of children's play and play environments: Toward a contemporary child-*

*saving movement*. Routledge.

Gerstein, E. D., & Crnic, K. A. (2018). Family interactions and developmental risk associated with early

cognitive delay: Influences on children’s behavioral competence. *Journal of Clinical Child & Adolescent Psychology*, *47*(sup1), S100-S112.

Gilboa, A., & Roginsky, E. (2010). Examining the dyadic music therapy treatment (DUET): The case of

a CP child and his mother*. Nordic Journal of Music Therapy, 1*9(2), 103-132.

Guralnick, M. J. (2017). Early intervention for children with intellectual disabilities: An update. *Journal*

*of Applied Research in Intellectual Disabilities*, *30*(2), 211-229.

Hamm, E.M. (2006). Playfulness and the environmental support of play in children with and without developmental disabilities. *OTJR; Occupation, Participation and Health, 26*(3), 88-96.

Himberg, T., & Thompson, M.R. (2011). Learning and synchronising dance movements in South African songs–cross-cultural motion-capture study. *Dance Research, 29*, 305-328.

Levavi, K., Menashe-Grinberg, A., Barak-Levy, Y., & Atzaba-Poria, N. (2020). The role of parental playfulness as a moderator reducing child behavioural problems among children with intellectual disability in Israel. *Research in Developmental Disabilities, 107*.

Mahony, G., Boyce, G., Fewell, R. R., Spiker, D., & Wheeden, C. A. (1998). The relationship of parent-

child interaction to the effectiveness of early intervention services for at-risk children and children with disabilities. *Topics in Early Childhood Special Education, 18*(1), 5–17.

Martin, A. M., O’Connor-Fenelon, M., & Lyons, R. (2010). Non-verbal communication between nurses

and people with an intellectual disability: a review of the literature. *Journal of Intellectual Disabilities, 14*(4), 303-314.

Menashe-Grinberg, A., & Atzaba-Poria, N. (2017). Mother–child and father–child play interaction: The

importance of parental playfulness as a moderator of the links between parental behavior and child negativity. *Infant Mental Health Journal, 38*(6), 772–784.

Norona, A. N., & Baker, B. L. (2017). The effects of early positive parenting and developmental delay

status on child emotion dysregulation. *Journal of Intellectual Disability Research, 61*(2), 130-143.

Okimoto, A. M., Bundy, A., & Hanzlik, J. (2000). Playfulness in children with and without disability: Measurement and intervention. *American Journal of Occupational Therapy, 54*(1), 73-82.‏

Patel, D. R., Cabral, M. D., Ho, A., & Merrick, J. (2020). A clinical primer on intellectual disability. *Translational Pediatrics, 9*, S23–S35.

Venuti, P., de Falco, S., Giusti, Z., & Bornstein, M. H. (2008). Play and emotional availability in young children with down syndrome. *Infant Mental Health Journal, 29*(2), 133–152.
