# Supplementary material for: ‘Just Play’ (JP) - creative arts therapies-based dyadic intervention for children with intellectual disability and their mothers: Study protocol for a mixed-methods randomized controlled trial
Source: PLoS One. 2026 May 21;21(5):e0349576. doi: 10.1371/journal.pone.0349576 (PMC13193551; doi:10.1371/journal.pone.0349576)
Supplement: Attachment — Submitted filename: Response to reviewers comments.docx [file pone.0349576.s004.docx]

Dear Editor,

We thank you and the reviewer for your careful review and constructive comments on our manuscript. We have addressed all points raised and revised the manuscript accordingly. We believe these revisions have improved the clarity and rigor of the paper and hope that the revised version is now suitable for publication in PLOS ONE.

Below, we provide a point-by-point response to each comment. The editor and reviewer’s comments are presented in black, and our responses are shown in blue. All revisions in the manuscript have been marked using track changes.

Sincerely,

Rita Abramov

on behalf of all authors

Editor comments

1. Please ensure that your manuscript meets PLOS ONE's style requirements, including those for file naming.

The manuscript has been revised in accordance with PLOS ONE’s style requirements.

2. PLOS ONE requires that all clinical trials are registered in an appropriate registry.

Please state the name of the registry and the registration number (e.g. ISRCTN or ClinicalTrials.gov) in the submission data and on the title page of your manuscript.
a) Please provide the complete date range for participant recruitment and follow-up in the methods section of your manuscript.
The name of the trial registry and the corresponding registration number have been added to the title page of the manuscript (ln 19-20). In addition, the Methods section has been revised to include the complete date range for participant recruitment and follow-up (ln 276-277).

3. PLOS ONE requires you to include in your manuscript further information about the funder so that any relevant competing interests can be assessed. This information should be included in your cover letter. We will amend your financial disclosure and competing interests on your behalf.

The updated cover letter includes the following information:

“The authors declare that they have no competing interests related to the funder. The funder had no role in study design, data collection and analysis, decision to publish, or preparation of the manuscript.”

4. Please provide a complete Data Availability Statement in the submission form, ensuring you include all necessary access information or a reason for why you are unable to make your data freely accessible. If your research concerns only data provided within your submission, please write "All data are in the manuscript and/or supporting information files" as your Data Availability Statement.

The updated submission form includes the following Data Availability Statement:

“All data regarding the study protocol are in the manuscript and supporting information files.”

5. We note that you have referenced (ie. Bewick et al. [5]) which has currently not yet been accepted for publication.

We were unable to identify the referenced citation in the current version of the manuscript.

6. Please include captions for your Supporting Information files at the end of your manuscript, and update any in-text citations to match accordingly

The manuscript has been revised to include captions for all Supporting Information files at the end of the document (ln 708-713).

7. We note that the original protocol that you have uploaded as a Supporting Information file contains an institutional logo. As this logo is likely copyrighted, we ask that you please remove it from this file and upload an updated version upon resubmission.

The institutional logos have been removed from S2_File, S3_File, S4_File, and S5_Files and updated versions have been uploaded accordingly.

8. In the current manuscript, major revision is required, especially for the introductory component emphasizing theories of play, the method clarifying randomization, and the discussion focusing on factual limitations.

We appreciate the Editor’s constructive comments. The manuscript has been thoroughly revised in response. The introduction now provides a more detailed theoretical framing of play (ln 67-108), the methods section clarifies the randomization procedure (ln 283-286), and the discussion more explicitly addresses the study’s limitations (ln 512-517). We believe these revisions have substantially strengthened the manuscript.

Reviewer comments

1. Introduction: The foundation of the study is the concept of play in developmental stages. More descriptions are needed. Key theorists should be mentioned to provide the readers with a clearer understanding of play. Additionally, more information is needed regarding the importance of play and its impact on children with intellectual disabilities (ID) and their mothers. What are the potential drawbacks if the ability to play is not adequately developed in children with ID?

Thank you for this important comment. The introduction has been revised to expand the theoretical and developmental understating of play, highlight key theorists, and discuss its significance for children with ID and their mothers (ln 67-108).

2. Could the authors explain why a psychoeducational parent counselling group was selected as the comparison group?

We have further elaborated on the selection of the psycho-educational parent counselling group as the comparison condition (ln 318-328).

3. Methods: Only the participants in the intervention group will undergo a 2-month follow-up assessment, whereas the participants in the control group will not be required to do so. This procedure is somewhat unusual. For a more robust design, both groups should be assessed at the same time point. Justification for this design is necessary.

A justification for conducting the 2-month follow-up assessment only in the intervention group has been added to the manuscript (ln 237-246).

4. Will the participants in the control group have the opportunity to participate in the JP intervention as part of a waitlist control design?

As the present study does not employ a waitlist control design, dyads allocated to the psycho-educational parent counselling condition will not be offered subsequent participation in the JP intervention within the framework of this trial. Furthermore, clarification on the study procedures has been added to the manuscript (ln 311-314).

5. While additional information has been provided regarding the design of the intervention, more details are needed, particularly in how the key elements of the JP intervention relate to the stages of play, and how these stages of play are connected to progress within the intervention.

The manuscript has been revised to provide additional details on how the key elements of the JP intervention correspond to the stages of play and how these stages relate to progress within the intervention (ln 332-341).

6. More descriptions are needed regarding the randomization and blinding procedures, such as who will be responsible for conducting these procedures and how the computer program operates.

The manuscript has been revised to provide expanded and clarified descriptions of both the randomization and blinding procedures (ln 283-289).

7. The sample size for the quantitative data analysis is well justified; however, a justification is needed for the sample size used in conducting the qualitative interviews. Furthermore, how will this subsample be recruited?

The manuscript has been revised to provide a more detailed description of the qualitative procedures, including the rationale for the sample size (ln 298-315).

8. Additional descriptions are necessary regarding the procedures for conducting qualitative interviews and the data analysis.

A Qualitative Data Analysis section has been added to describe the procedures for analyzing the qualitative study data (ln 476-494).

9. Where will the participants be recruited from? Since children with autism spectrum disorder (ASD) will be excluded from the study, how can the authors ensure that they will recruit a sufficient number of participants within the study period, given the high comorbidity between ASD and ID?

Thank you for this comment regarding the current clinical reality. The manuscript has been revised to clarify the participant recruitment procedures (ln 257-258).

10. Discussion: The limitations section should include the fact that the participants are not blinded to the group condition.

The manuscript has been revised to explicitly address the lack of participant blinding in addition to other limitation (ln 512-517).
