# Supplementary material for: ‘Just Play’ (JP) - creative arts therapies-based dyadic intervention for children with intellectual disability and their mothers: Study protocol for a mixed-methods randomized controlled trial
Source: PLoS One. 2026 May 21;21(5):e0349576. doi: 10.1371/journal.pone.0349576 (PMC13193551; doi:10.1371/journal.pone.0349576)
Supplement: Attachment — Submitted filename: Response to the reviewers comments.docx [file pone.0349576.s005.docx]

Dear Reviewer

We thank you for your careful review and constructive comments on the statistical analysis in our manuscript. We have addressed all points raised and revised the manuscript accordingly. We believe these revisions have improved the clarity and rigor of the paper and hope that the revised version is now suitable for publication in PLOS ONE.

Below, we provide a point-by-point response to each comment. The reviewer’s comments are presented in black, and our responses are shown in blue. All revisions in the manuscript have been marked using track changes.

Sincerely,

Rita Abramov

on behalf of all authors

(a) The primary analysis plan is too simplistic for the number and structure of outcomes. A two-way repeated-measures ANOVA for EA and IPS only, with Time = pre/post and Condition = intervention/control, was proposed. However, it's not clear, if each EA dimension (EA has multiple subscales) will be analyzed separately. Will multiplicity be controlled. Also, it's not clear, what would be the "primary outcome".

(b) Again, the sample size is based on change in EA scales, but it's not clear (to me) that the EA is the primary outcome variable.

Thank you for these comments. We have now explicitly designated the EA total sum score as the primary outcome for this study, as well as changing the analytical method, thereby reducing the risk of multiplicity (ln 466-471).

(c) The analytical method - 2-way repeated measures ANOVA, may not be adequate in presence of dropouts/missing data. Alternative linear mixed models, with a missing data imputation technique embedded, appears more elegant. On the overall, the methodology for handling missing data is not clearly written.

To address dropout and potential missing data, we will use **Linear Mixed-Effects Models (LMM)** using a Restricted Maximum Likelihood (REML) approach, which includes time, group, and their interaction effects. Given that all effects were tested within a single pre-specified model, no correction for multiplicity will be applied, consistent with recommendations in the statistical literature (e.g., Rothman, 1990; Fitzmaurice et al., 2011) (ln 466-471).

(d) The mediation analysis plan appeared too optimistic, mostly due to the small sample size issue in this kind of small randomized trials, as well as modeling simple change scores.

We have addressed this important comment and revised the mediation analysis plan. Given our modest sample size (n=60), the mediation analyses should be considered exploratory and interpreted with caution. To reduce the risk of overfitting, only predictors with theoretical (treatment group, child’s VABS-3 score) and statistical justification will be included in the model, and indirect effects will be estimated using bootstrap confidence intervals (ln 471-483).

(e) In randomized trials, covariate adjustment should be based on clinical relevance or pre-specification, not on baseline significance tests, which are notoriously unhelpful and sample-size dependent; read here: <https://pubmed.ncbi.nlm.nih.gov/12325108/>
Hence, comments like: "If any demographic variable differs significantly between groups, it will be entered as a covariate in the ANOVA models." are misleading.

We thank the reviewer for this important comment. We have changed the analysis so the covariates will be adjusted based on clinical relevance: the child’s gender and their VABS-3 score (ln 480-483).
